# Miniaturized Multi-Cantilever MEMS Resonators with Low Motional Impedance

**DOI:** 10.3390/mi15060688

**Published:** 2024-05-24

**Authors:** Haolin Li, Qingrui Yang, Yi Yuan, Shuai Shi, Pengfei Niu, Quanning Li, Xuejiao Chen, Menglun Zhang, Wei Pang

**Affiliations:** State Key Laboratory of Precision Measuring Technology and Instruments, Tianjin University, Tianjin 300072, China

**Keywords:** aluminum nitride, low motional impedance, piezoelectric actuated cantilever, mechanically coupled multi-cantilever, double-sided actuation

## Abstract

Microelectromechanical system (MEMS) cantilever resonators suffer from high motional impedance (*R_m_*). This paper investigates the use of mechanically coupled multi-cantilever piezoelectric MEMS resonators in the resolution of this issue. A double-sided actuating design, which utilizes a resonator with a 2.5 μm thick AlN film as the passive layer, is employed to reduce *R_m_*. The results of experimental and finite element analysis (FEA) show agreement regarding single- to sextuple-cantilever resonators. Compared with a standalone cantilever resonator, the multi-cantilever resonator significantly reduces *R_m_*; meanwhile, the high quality factor (*Q*) and effective electromechanical coupling coefficient (*Kt_eff_*^2^) are maintained. The 30 μm wide quadruple-cantilever resonator achieves a resonance frequency (*f_s_*) of 55.8 kHz, a Q value of 10,300, and a series impedance (*R_s_*) as low as 28.6 kΩ at a pressure of 0.02 Pa; meanwhile, the smaller size of this resonator compared to the existing multi-cantilever resonators is preserved. This represents a significant advancement in MEMS resonators for miniaturized ultra-low-power oscillator applications.

## 1. Introduction

Reference oscillators are essential components that are widely employed in various electronics systems to provide frequency control and fulfill timing requirements [1]. With the development of real-time clocks and the Internet of Things (IoT), the application of miniaturized ultra-low-power oscillators is essential [2]. In the design of oscillators, low-frequency resonators are the preferred choice because the use of higher-frequency oscillators results in a significant increase in current consumption by several orders of magnitude [3,4]. Quartz tuning fork resonators vibrating at 32,768 Hz are the main technology used in low-frequency resonators due to their high quality factor (*Q*) and intrinsic level of stability [5]. However, it is difficult to further reduce the size of quartz tuning fork resonators, which results in the dramatic degradation of *Q* and an increase in the motional impedance (*R_m_*) [6,7]. In the case of one-port resonators operating within a sustained oscillation loop, *R_m_* and Q influence the power consumption, close-to-carrier phase noise, and zero-phase oscillation conditions.

By contrast, MEMS resonators have intrinsic advantages with regard to their capacity to be miniaturized [8]. Compared with capacitive MEMS resonators, piezoelectric resonators have a higher electromechanical coupling factor [9,10] and thus lower motional impedance [11,12], which is beneficial to the development of ultra-low-power oscillators. However, their conventional stand-alone cantilever structure is an obstacle to the attainment of further reductions in the motional impedance via an increase in the resonator area. Once the resonant frequency of the cantilever resonator is determined, the length-to-thickness ratio of the resonator is held constant [5]. Consequently, elongating the length of the resonator to increase its area would lead to a power series expansion in thickness, thereby exerting pressure on the fabrication process. On the other hand, widening the cantilever resonator would result in an increase in the thermoelastic damping loss and anchor loss [13,14], which in turn would diminish the resonator’s *Q*.

In this paper, the low motional impedance of mechanically coupled, double-sided actuating multi-cantilever piezoelectric MEMS resonators is investigated. While using multiple cantilevers is straightforward in principle, the coupled system exhibits complex behavior that modulates key parameters, such as the effective electromechanical coupling coefficient (*Kt_eff_*^2^) and spurious modes, which will be discussed later. Microcantilevers of varying numbers and with different vibrational arm widths are designed, fabricated, and characterized. The quadruple cantilever proves to be a resonator structure that can achieve a low *R_m_*, high *Kt_eff_*^2^, and compact dimensions.

## 2. Device Design

The *R_m_* of the resonator can be expressed as follows [15]:

(1) $$R_{m}\propto \frac{1}{f_{s}C_{0}Kt_{eff}^{2}Q}$$

In Equation (1), *f_s_*, *C*_0_, *Kt_eff_*^2^, and *Q* represent the series resonance frequency, static capacitance, effective electromechanical coupling coefficient, and quality factor, respectively. It is evident that the performance of *R_m_* is not isolated but influenced by other performance parameters. The primary method used to reduce *R_m_* involves increasing the resonator’s *C*_0_. For multi-cantilever resonators, the area of the resonator is N times that of a single cantilever, where N denotes the number of arms. Consequently, the *C*_0_ of the resonator increases by a factor of N, ideally reducing the *R_m_* of a multi-cantilever beam resonator to 1/N of that for a single cantilever. Equation (1) also indicates that *Kt_eff_*^2^ and *Q* should not be compromised with the multi-cantilever beam resonator design for reduced *R_m_*.

**Figure 1:**
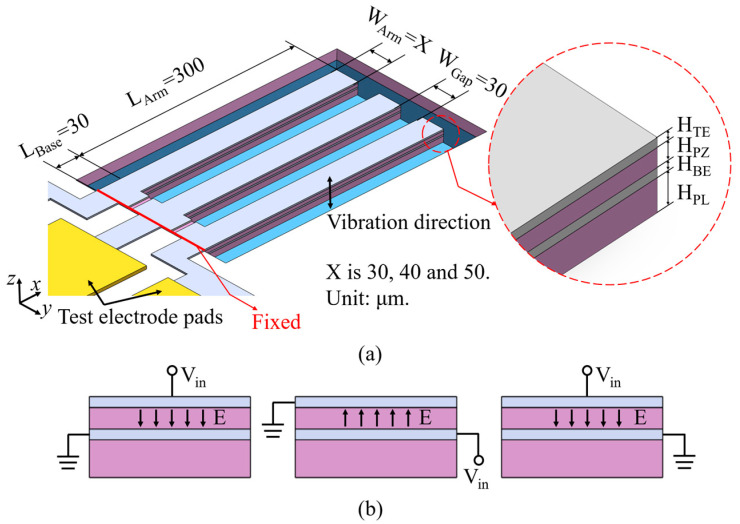
Schematic of a triple-cantilever resonator with (**a**) typical geometric parameters and (**b**) electrical signal excitation.

**Figure 2:**
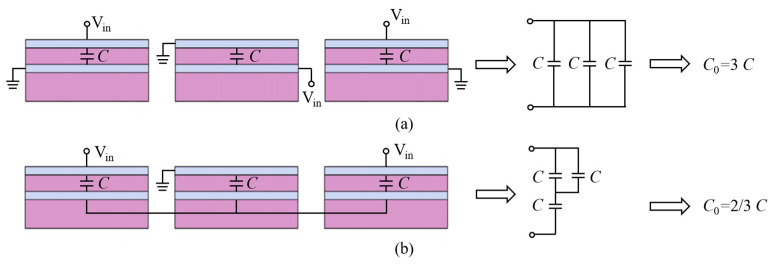
Equivalent *C*_0_ of the triple-cantilever resonator for (**a**) double-sided actuation configuration and (**b**) single-sided actuation configuration.

The multi-cantilever resonator comprises a base and arms, with all arms connected to the base, and the base affixed to the structure. As shown in Figure 1a, the triple-cantilever resonator is featured as an example, and the other multi-cantilever resonators have arms with identical dimensions and gaps. The resonator stack, from bottom to top, includes a passive layer (PL), a bottom electrode (BE), a piezoelectric layer (PZ), and a top electrode (TE). Figure 1b illustrates the method used to connect the electrode in the triple-cantilever resonator; the BE and TE of each arm are connected to signal or ground terminals, respectively, and electrodes carrying the same signal are connected to identical test electrode pads. As shown in Figure 2, compared to the single-sided actuation configuration with a floating bottom electrode, the double-sided actuation configuration serves to increase the resonator’s *C*_0_, thereby reducing its *R_m_*.

The *R_m_* of cantilever-type resonators typically lies in the kiloohm range, similar to the impedance of the silicon substrate. This precludes treating the silicon substrate as an insulating substrate, and thus a consideration of its impedance properties is necessitated. Therefore, an insulating layer (passive layer) between the BE and the substrate is required to ensure isolation. In addition, to ensure compatibility with the fabrication process, this study employs an aluminum nitride (AlN) thin film as both the piezoelectric layer and passive layer to achieve an out-of-plane flexural resonant mode.

**Figure 3:**
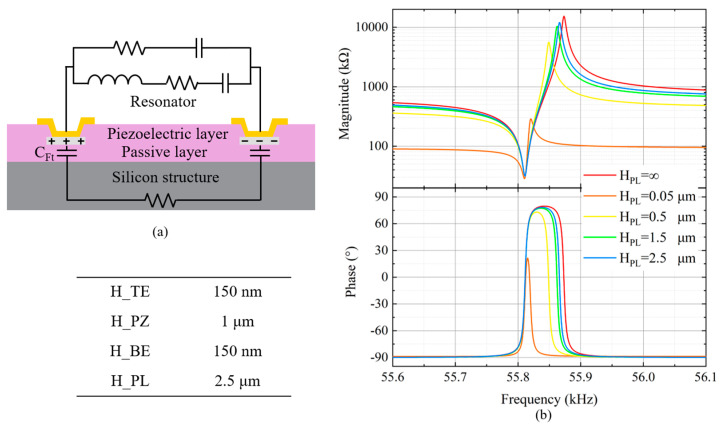
(**a**) Illustration of the parasitic capacitance and resistance induced by the passive layer and silicon substrate. (**b**) Simulated frequency response curves of resonators with passive layers of different thicknesses.

Due to the presence of the AlN passive layer in the resonator, there is a parasitic capacitance (*C*_Ft_) that exists in parallel with the resonator’s test electrode pads, as illustrated in Figure 3a. The thickness of the passive layer inversely affects this parasitic capacitance; thinner layers result in higher capacitance, which severely degrades the resonator’s performance. This degradation is evidenced by a reduction in the parallel impedance (*R_p_*) and a decrease in the *Kt_eff_*^2^, along with phase distortion. Figure 3b demonstrates how varying thicknesses of the AlN passive layer influence the resonator’s performance. Considering that using an AlN with an excessive thickness can complicate the fabrication process, this study employs passive and piezoelectric layers of AlN with thicknesses of 2.5 μm and 1 μm, respectively. At this thickness, the *Kt_eff_*^2^ of the resonator achieves 88.5% of the performance that is possible with an infinitely thick passive layer. The calculation formula for the *Kt_eff_*^2^ is as follows:

(2) $$Kt_{eff}^{2}=\frac{\frac{\pi }{2}\frac{f_{s}}{f_{p}}}{\tan\left(\frac{\pi }{2}\frac{f_{s}}{f_{p}}\right)}$$

In Equation (2), *f_s_* and *f_p_* represent the series and parallel resonance frequencies, respectively. The table in Figure 3 presents the dimensional parameters for the stack of the resonator, where both the top and bottom electrode materials are molybdenum (Mo).

To balance the torque at the base, the electrical polarity of each arm is symmetrically arranged along the resonator centerline, with adjacent arms in half of the resonator displaying inverse electrical polarity. When arms with the opposite polarity vibrate in the opposite direction, the main mode is excited. Conversely, if the arms with opposite electrical polarities vibrate in the same direction and the amplitudes of the arms differ, the partial cancellation of the piezoelectrically induced charge results in the generation of a spurious resonant response.

**Figure 4:**
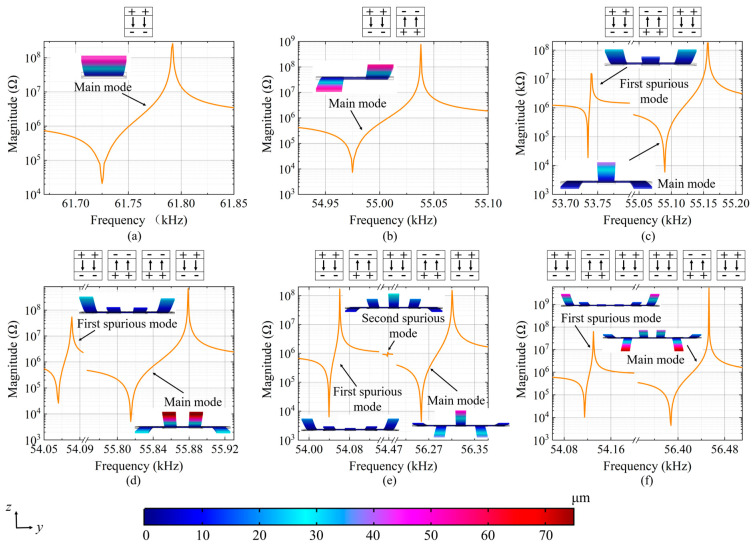
Frequency response curves and respective mode shapes obtained from finite element simulations of the (**a**) single-, (**b**) double-, (**c**) triple-, (**d**) quadruple-, (**e**) quintuple-, and (**f**) sextuple-cantilever resonators with a width of 30 μm.

Figure 4 displays the simulated frequency response curves obtained for single- to sextuple-cantilever resonators with a width of 30 μm, alongside 3D finite element analysis (FEA) images illustrating the main mode and the spurious mode of vibration. These figures also show the arrangement of signals for the top and bottom electrodes in each image, where “+” and “−” represent the signal and ground terminals, respectively. Due to this arrangement, when two adjacent arms vibrate in the same direction, the piezoelectrically induced charge on the electrode surface provides counterbalance. It can be seen that the spurious mode is observed from the triple-cantilever resonator to the sextuple-cantilever resonator.

**Figure 5:**
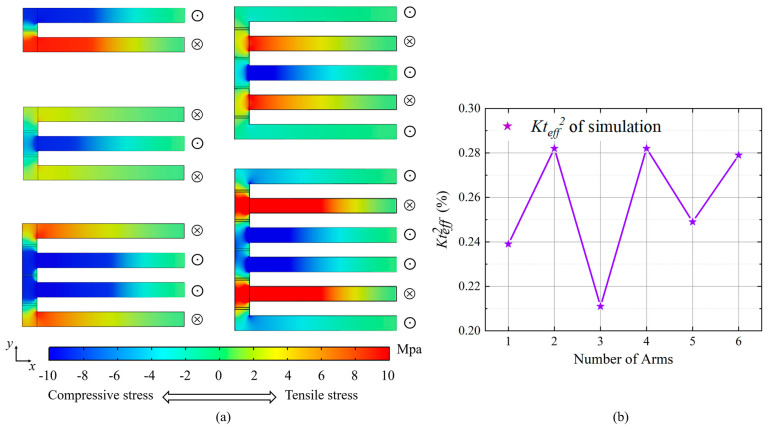
(**a**) Diagrams of the stress distribution on the xy plane for the main modes of multi-cantilever resonators (⊙ and ⊗ denote the upward and downward vibrations of the corresponding arm, respectively). (**b**) *Kt_eff_*^2^ calculation for multi-cantilever resonators using finite element simulations.

In comparison with single-cantilever resonators, the vibrations of each arm in multi-cantilever resonators are mechanically coupled through the base. Diagrams of the stress distribution on the xy plane of the upper surface for the main modes of the resonators with double- to sextuple-cantilever resonators are exhibited in Figure 5a. It is evident that double-, quadruple- and sextuple-cantilever resonators experience greater stress at the base. The *Kt_eff_*^2^ for multi-cantilever resonators, which is calculated using the amplitude–frequency characteristic curves obtained from finite element simulations, is presented in Figure 5b. The results show that the double-, quadruple- and sextuple-cantilever resonators have a higher *Kt_eff_*^2^. Based on the foregoing FEA, it is observable that, under identical actuating voltages, the higher the stress at the resonator’s base, the greater the *Kt_eff_*^2^ of the resonator. We speculate that the stress at the base modulates the coupling between the electrical and mechanical domains of the multi-cantilever resonators, which will be further investigated in our future research. Due to the varying number of arms, the *Kt_eff_*^2^ differs among multi-cantilever resonators. Consequently, the *R_m_* in Equation (1) does not decrease in strict accordance with the multiple relations.

## 3. Device Fabrication

**Figure 6:**
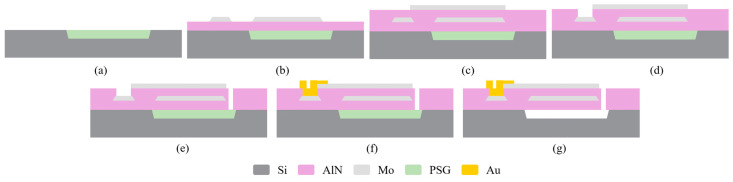
Process used to fabricate the multi-cantilever resonators. (**a**) Etching the Si wafer and filling it with PSG. (**b**) Sputtering deposition of the AlN as a structural layer and the deposition and patterning of the Mo as the bottom electrode. (**c**) Sputtering deposition of the AlN as the piezoelectric layer and the deposition and patterning of the Mo as the top electrode. (**d**) Etching of 1 μm of AlN. (**e**) Etching of 3.5 μm of AlN. (**f**) Deposition and patterning of the Au layer. (**g**) Release of resonator in HF solution.

**Figure 7:**
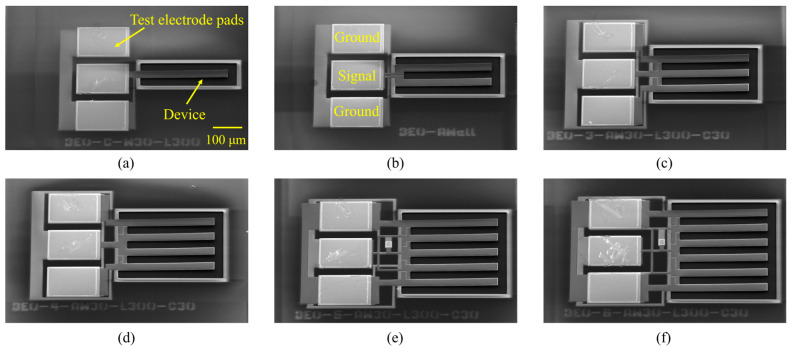
SEM images of the (**a**) single-, (**b**) double-, (**c**) triple-, (**d**) quadruple-, (**e**) quintuple-, and (**f**) sextuple-cantilever resonators with a width of 30 μm.

Figure 6 shows a simplified fabrication process. First, an air cavity is etched directly onto a silicon wafer using reactive-ion etching and then filled with phosphosilicate glass (PSG) as a sacrificial layer, as shown in Figure 6a. A total of 2.5 μm of AlN is deposited and used as the passive layer via RF sputtering. Then, 150 nm of Mo is also deposited and patterned as the bottom electrode via plasma etching, as shown in Figure 6b. Next, 1 μm of AlN is deposited and used as the piezoelectric layer. Then, 150 nm of Mo is deposited and patterned as the top electrode, as shown in Figure 6c. Next, 1 μm of AlN is etched via Cl_2_-based plasma etching and potassium hydroxide (KOH) wet etching; this exposes the bottom electrode, as shown in Figure 6d. Afterward, 3.5 μm of AlN is etched via Cl_2_-based plasma etching; this forms the shape of the resonant cavity, as shown in Figure 6e. To connect the bottom and top electrodes, 600 nm of Au is deposited via physical vapor deposition and is patterned based on the lift-off, as shown in Figure 6f. Finally, the PSG is released in the HF solution, as shown in Figure 6g. Figure 7 shows scanning electron microscopy (SEM) images of the fabricated multi-cantilever resonators with a width of 30 μm.

## 4. Measurement Results

**Figure 8:**
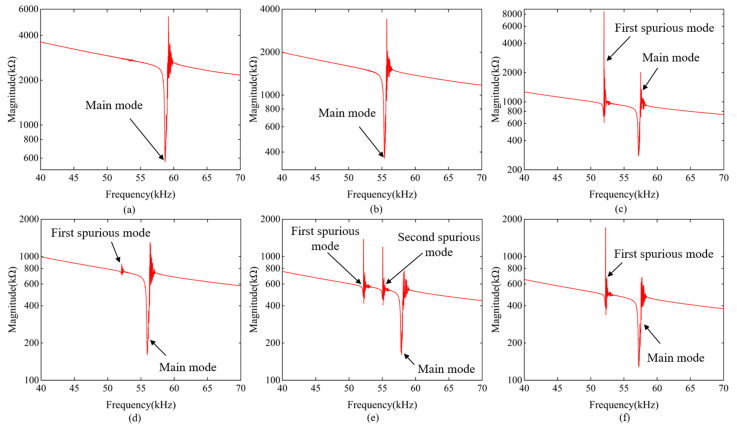
Measured impedance magnitude curves of the (**a**) single-, (**b**) double-, (**c**) triple-, (**d**) quadruple-, (**e**) quintuple-, and (**f**) sextuple-cantilever resonators with a width of 30 μm.

The frequency response measurements for the single- to sextuple-cantilever resonators with a width of 30 μm in a vacuum environment are presented in Figure 8. For one-port measurements, probes were connected to test pads and an impedance analyzer (E4990A, Keysight Technologies, USA). The spurious and main modes observed in the measured frequency response curves are consistent with the finite element simulation results presented in Figure 4. It should be noted that the curve near the resonance of each parallel resonator appears unsmooth. This is an artifact that arises when the scanning frequency range is broad. The artifacts come from the impedance analyzer we used when spanning frequency is large. When the scanning frequency range is reduced, the frequency interval is accordingly reduced and these oscillations around the *f_p_* disappear, as shown in Figure 10a.

**Figure 9:**
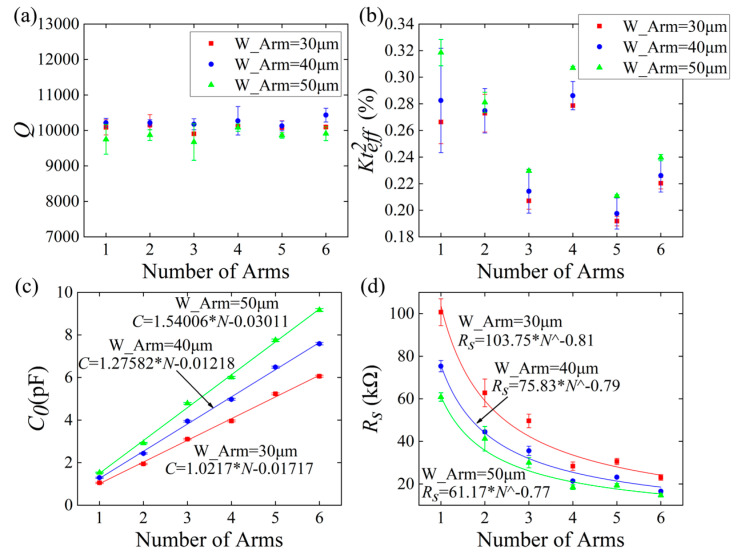
Measurement results for (**a**) *Q*, (**b**) *Kt_eff_*^2^, (**c**) *C*_0_ and (**d**) *R_s_* of the single- to sextuple-cantilever resonators with W_Arm values of 30 μm, 40 μm and 50 μm.

Figure 9 presents a scatter distribution of *Q* and *Kt_eff_*^2^, along with a fitting curve that depicts the variations in *C*_0_ and the series impedance (*R_s_*, the impedance at the series resonant frequency, which directly reflects the level of *R_m_*), as measured when the W_Arm value of the single- to sextuple-cantilever resonators is set to 30 μm, 40 μm, and 50 μm. As shown in Figure 9a, *Q* is randomly distributed at approximately 10,000, indicating that neither the number of arms nor the variation in width established in this study significantly affect the Q value of the resonator. However, both the number and width of the arms influence *Kt_eff_*^2^, as illustrated in Figure 9b. In the trends in *Kt_eff_*^2^ that can be observed, the measurements align closely with the simulation trends depicted in Figure 5b. Notably, the *Kt_eff_*^2^ values measured for the sextuple-cantilever resonators are lower than those predicted via the finite element simulations. This discrepancy can be attributed to the increased complexity of the external electrode leads, which is caused by the greater number of arms; in turn, this deteriorates the *Kt_eff_*^2^.

When W_Arm remains constant, *C*_0_ exhibits a linear relationship with the number of arms, as shown in Figure 9c. In addition, *R_s_* is inversely proportional to the number of arms, as depicted in Figure 9d. Nevertheless, the *R_s_* value of quintuple-cantilever resonators is slightly higher than that of quadruple-cantilever resonators. The reason for this is that the *Kt_eff_*^2^ value of quintuple-cantilever resonators is significantly smaller than that of other resonators. When the number of cantilevers exceeds four, the downward trend observed in *R_s_* becomes less pronounced; meanwhile, there is a significant increase in area, which is disadvantageous regarding the miniaturization of MEMS devices. Additionally, quadruple-cantilever resonators exhibit a *Kt_eff_*^2^ comparable to that of single- and double- cantilever resonators. Therefore, quadruple-cantilever resonators represent the best multi-cantilever resonator design, combining high electromechanical coupling and low motional impedance to achieve superior resonator performance.

## 5. Discussion

Table 1 compares the performance of the quadruple-cantilever resonator proposed in this study with resonators that have previously been developed and feature piezoelectric AlN out-of-plane flexural modes; the comparison parameters include *Q*, *R_s_*, *Kt_eff_*^2^, *f* × *Q*, and the figure of merit (FoM). Resonators with a high Q enhance the frequency stability and phase noise of oscillators and contribute to an improved resolution and signal-to-noise ratio. A low *R_s_* improves the signal-to-noise ratio and reduces the energy consumption of oscillators. Additionally, a large *Kt_eff_*^2^ provides a broader range of excitation frequencies for oscillators. Furthermore, for miniaturized MEMS devices, the size of the components is significant; hence, the area of the resonators has also been compared. The resonator introduced in this research exhibits an enhanced overall performance regarding the *f* × *Q* product and FoM compared to prior studies. Its performance is nearly comparable to that of devices developed by Murata Manufacturing Co., Ltd. [16], but it offers added advantages; these include a significantly lower *R_s_* and a resonator size that is only one-fifth of that of Murata’s corresponding chip dimensions.

**Figure 10:**
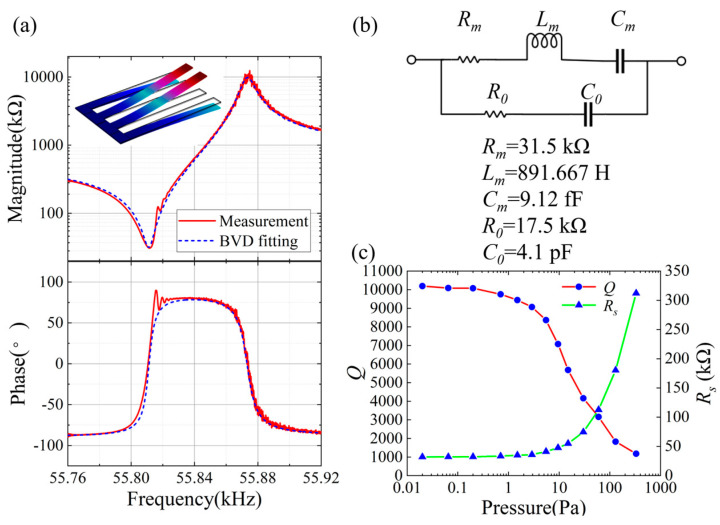
Detailed measurement results for a quadruple-cantilever resonator with a W_Arm value of 30 μm: (**a**) frequency response and mBVD model fitting curves, (**b**) the equivalent circuit parameters of the mBVD model, and (**c**) pressure dependence of *Q_mBVD_* and *R_s_*.

Figure 10a shows the electrical properties of the main mode for the smallest quadruple-cantilever resonator designed in this study, which features a W_Arm value of 30 μm, measured at a pressure of 0.02 Pa. The measured 3 dB quality factor (*Q_Mea_*) is 10,300, with an *R_s_* of 28.6 kΩ at an *f_s_* of 55.8 kHz. The corresponding parameters of the modified Butterworth–van Dyke (mBVD) model are displayed in Figure 10b, which shows a fitted quality factor (*Q_mBVD_*) of 10,175.

Figure 10c presents the measured pressure dependence of both *Q_Mea_* and *R_s_* for the main mode of the resonator. When the pressure is below 5 Pa, the *Q_Mea_* of the resonator is able to maintain over 83% of its maximum value; this indicates that the resonator operates within the “intrinsic region” [7]. At this pressure range, *R_s_* does not decrease significantly.

## 6. Conclusions

In summary, this study evaluates the design of multi-cantilever MEMS resonators that can reduce *R_m_* and maintain miniaturization through experimental analysis and FEA. The influence of the number and size of the cantilevers on the *Q*, *Kt_eff_*^2^, *C*_0_, and *R_s_* values of the main mode was examined. To maintain a compact size, a 30 μm wide quadruple-cantilever resonator with an *f_s_* of 55.8 kHz, a *Q* value of 10,300, and *R_s_* of 28.6 kΩ at 0.02 Pa was preferred.

## Figures and Tables

**Table 1:** Comparison of performance with reported piezoelectric AlN out-of-plane flexural mode resonators in a vacuum.

Structure	Frequency (kHz)	*Q*	*R_s_* (kΩ)	*Kt_eff_*^2^ (%)	*f* × *Q* (×10^7^)	FoM ^1^	Area (×10^4^ μm^2^)	Reference
Cantilever	7	7500	N/A	N/A	5.3	N/A	N/A	[17]
Cantilever	19.6	3320	N/A	0.13	6.5	4.3	N/A	[18]
Cantilever	50.1	4328	N/A	N/A	22	N/A	N/A	[19]
Tuning fork (in-phase)	61.38	2799	N/A	N/A	17	N/A	N/A	[20]
Tuning fork (out of phase)	124.99	3480	N/A	N/A	43	N/A	N/A	[20]
Triple-cantilever	70	4000	N/A	0.21	28	8.4	100	[21]
Quadruple-cantilever	32	20,000	50	0.15	64	30	54 ^2^	[16]
Quadruple-cantilever (W_Arm = 30 μm)	55.8	10,300	28.6	0.26	57	26.8	9.7	This work
Quadruple-cantilever (W_Arm = 50 μm)	53.6	10,100	19.2	0.31	54	31.3	12.6	This work

^1^ FoM is the product of *Kt_eff_*^2^ and *Q*. ^2^ Chip-scale package dimensions.

## Data Availability

The data presented in this study are available from the corresponding author on request. The data are not publicly available due to privacy.
